# Costs and effects of a state-wide health promotion program in primary schools in Germany – the Baden-Württemberg Study: A cluster-randomized, controlled trial

**DOI:** 10.1371/journal.pone.0172332

**Published:** 2017-02-21

**Authors:** Dorothea Kesztyüs, Romy Lauer, Tibor Kesztyüs, Reinhold Kilian, Jürgen M Steinacker, on behalf of the “Join the Healthy Boat” Study Group

**Affiliations:** 1 Section Sports and Rehabilitation Medicine, Department of Internal Medicine II, Ulm University, Ulm, Germany; 2 Institute of General Medicine, Ulm University, Ulm, Germany; 3 Department of Computer Science, Ulm University of Applied Sciences, Ulm, Germany; 4 Section Health Economics and Health Services Research, Department of Psychiatry II, Ulm University, Günzburg, Germany; University of Washington, UNITED STATES

## Abstract

**Aim:**

To evaluate the cost-effectiveness of the state-wide implementation of the health promotion program “Join the Healthy Boat” in primary schools in Germany.

**Methods:**

Cluster-randomized intervention trial with wait-list control group. Anthropometric data of 1733 participating children (7.1 ± 0.6 years) were taken by trained staff before and after a one year intervention period in the academic year 2010/11. Parents provided information about the health status, and the health behaviour of their children and themselves, parental anthropometrics, and socio-economic background variables. Incidence of abdominal obesity, defined as waist-to-height ratio (WHtR) ≥ 0.5, was determined. Generalized linear models were applied to account for the clustering of data within schools, and to adjust for baseline-values. Losses to follow-up and missing data were analysed. From a societal perspective, the overall costs, costs per pupil, and incremental cost-effectiveness ratio (ICER) to identify the costs per case of averted abdominal obesity were calculated.

**Results:**

The final regression model for the incidence of abdominal obesity shows lower odds for the intervention group after an adjustment for grade, gender, baseline WHtR, and breakfast habits (odds ratio = 0.48, 95% CI [0.25; 0.94]). The intervention costs per child/year were €25.04. The costs per incidental case of averted abdominal obesity varied between €1515 and €1993, depending on the different dimensions of the target group.

**Conclusion:**

This study demonstrates the positive effects of state-wide, school-based health promotion on incidental abdominal obesity, at affordable costs and with proven cost-effectiveness. These results should support allocative decisions of policymakers. An early start to the prevention of abdominal obesity is of particular importance because of its close relationship to non-communicable diseases.

**Trial registration:**

German Clinical Trials Register (DRKS), Freiburg University, Germany, DRKS-ID: DRKS00000494.

## Introduction

As a result of the globalization of unhealthy lifestyles, characterized by poor diet and physical inactivity, obesity is one of the most discussed risk factors for non-communicable diseases (NCDs) worldwide [1]. These diseases are currently the world’s main killers, totalling 63% of all deaths. The economic burden of NCDs is substantial. Macroeconomic simulations suggested a cumulative output loss of 75% of global gross domestic product (GDP) in 2010 [1]. Several NCDs are associated with the metabolic syndrome, in which abdominal obesity is the most obvious and prevalent constituent [2]. Rates of abdominal obesity are rising; although some researchers report a levelling off, or plateauing of obesity, as defined by body mass index (BMI) [3, 4, 5]. Weight gain most often starts in early childhood and frequently persists into adulthood [6, 7]. Many obese children already show metabolic complications and are at high risk for the development of early morbidity [8]. Timely initiation of evidence-based prevention and health promotion addressing children, parents, caregivers, and teachers is urgently needed to reverse the trend.

Programs for health promotion and prevention in the school setting are ubiquitous, and some have proven their effectiveness [9]. With regard to limited resources, it is essential to learn more about the cost-effectiveness of those programs and interventions.

In their extensive systematic Cochrane review on interventions for preventing obesity in children in 2011, Waters and colleagues did not find any study that included a formal economic evaluation [9]. In 2014, Langford and colleagues found only two studies on cost-effectiveness in their Cochrane review of the WHO health promoting school framework [10]. John and colleagues, who focused especially on the cost-effectiveness of interventions on paediatric obesity, found less than 10 studies on preventive measures in 2012 [11]. One of the studies, presented in both reviews by Langford as well as by John, was the “Ulm Research on Metabolism, Exercise and Lifestyle Intervention in Children” (URMEL-ICE). Assuming a parental willingness to pay €35/year, the URMEL-ICE intervention was cost-effective in preventing children from an increase in abdominal girth [12]. Based on this positive result, the intervention mentioned was revised, and renamed as the “Join the Healthy Boat” health promotion program; it spread throughout the state of Baden-Württemberg in southern Germany. Health economic results from the outcome evaluation of this “Baden-Württemberg Study” shall be presented here.

### Aims

The purpose of the present study was to assess the cost-effectiveness of a school-based, state-wide, health promotion program. Therefore, the costs were assessed in detail, calculated in total and per capita, and were compared to the number of averted cases of incidental abdominal obesity.

## Participants and methods

### Study description

The “Baden-Württemberg Study” was the outcome evaluation of the school-based, health promotion program, “Join the Healthy Boat”, and was designed as a cluster-randomized, controlled intervention trial. The stratified cluster-randomization process resulted in an approximately balanced randomization [13]. Blinding was not feasible. Sample size calculations were based on available data for the primary outcome waist circumference (WC) [13]. The primary outcome measures for the health economic analysis were longitudinal changes in WC and waist-to-height ratio (WHtR).

The study was conducted throughout the state of Baden-Württemberg, which is located in southern Germany. The intervention group was comprised of schools where teachers had successfully completed the vocational training and implemented the “Join the Healthy Boat” intervention in the academic year 2010/2011. The teachers in the schools of the control group, representing the alternative in the economic analysis, continued to teach as normal and were obliged to wait one year before they could take part in the vocational training. Approval from the Ethics Committee of Ulm University and written informed consent from parents was obtained. The study was registered on the German Clinical Trials Register (DRKS), Freiburg University, Germany, under the DRKS-ID: DRKS00000494. Detailed information concerning the trial has already been published elsewhere [13].

### Intervention

The health promotion program, “Join the Healthy Boat”, developed by the scientific researchers of Ulm University, targets the healthy lifestyle of primary school children in grades 1 to 4, and also focuses on the prevention of children becoming overweight and obese. To guarantee systematic and evidence-based development, the evaluation and implementation of the program, the intervention mapping approach, [14] as well as the social cognitive theory [15] and the socio-ecological model [16], were utilized. The three main topics of the program are the promotion of physical activity, the reduction in intake of sugar-sweetened beverages and the reduction of screen media consumption. All intervention materials were integrated into the regular curriculum; no extra lessons were required. As well as course materials for the teachers, the intervention materials include materials for children (e.g. activity breaks) and for parents (e.g. family homework and information material).

In order to ensure the state-wide implementation of the program, a “train-the-trainer” concept was applied. For this purpose, 32 experienced teachers, spread all over Baden-Württemberg, were trained intensively in the concept and the materials, and attended seminars, provided by the scientific staff of Ulm University, twice a year. Those teachers, further referred to as “consulting teachers”, in turn, trained teachers in their regions (“peer-to-peer”) in three vocational training sessions, in order to provide them with the necessary knowledge and practical skills to apply the program in their classes. More detailed information about the intervention, the data collection, the randomization process, and the sample size calculation can be found in the published study protocol, S1 Protocol,[13].

### Participants

**Fig 1:**
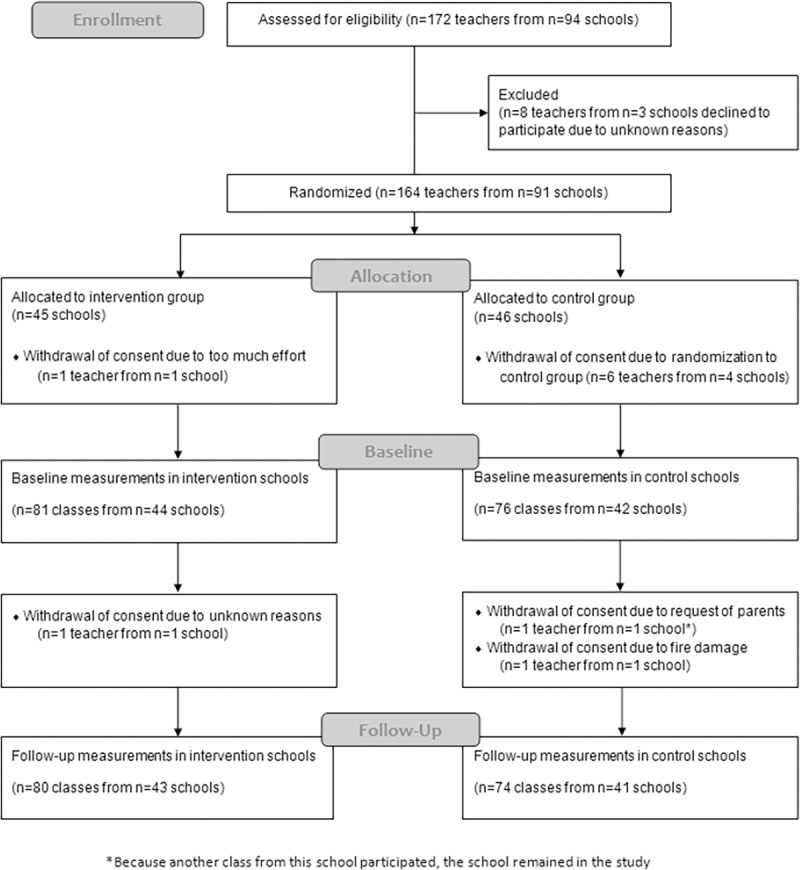
Flowchart of enrollment, baseline measurements, and follow-up of teachers, classes and schools in the Baden-Württemberg Study. Adapted from “Evaluation of a health promotion program in children: Study protocol and design of the cluster-randomized Baden-Wuerttemberg primary school study [DRKS-ID: DRKS00000494].,” by Dreyhaupt J, Koch B, Wirt T, Schreiber A, Brandstetter S, Kesztyues D, et al. BMC Public Health. 2012;12(1):157. Copyright 2012 by Dr. Jens Dreyhaupt. Adapted with permission.

**Fig 2:**
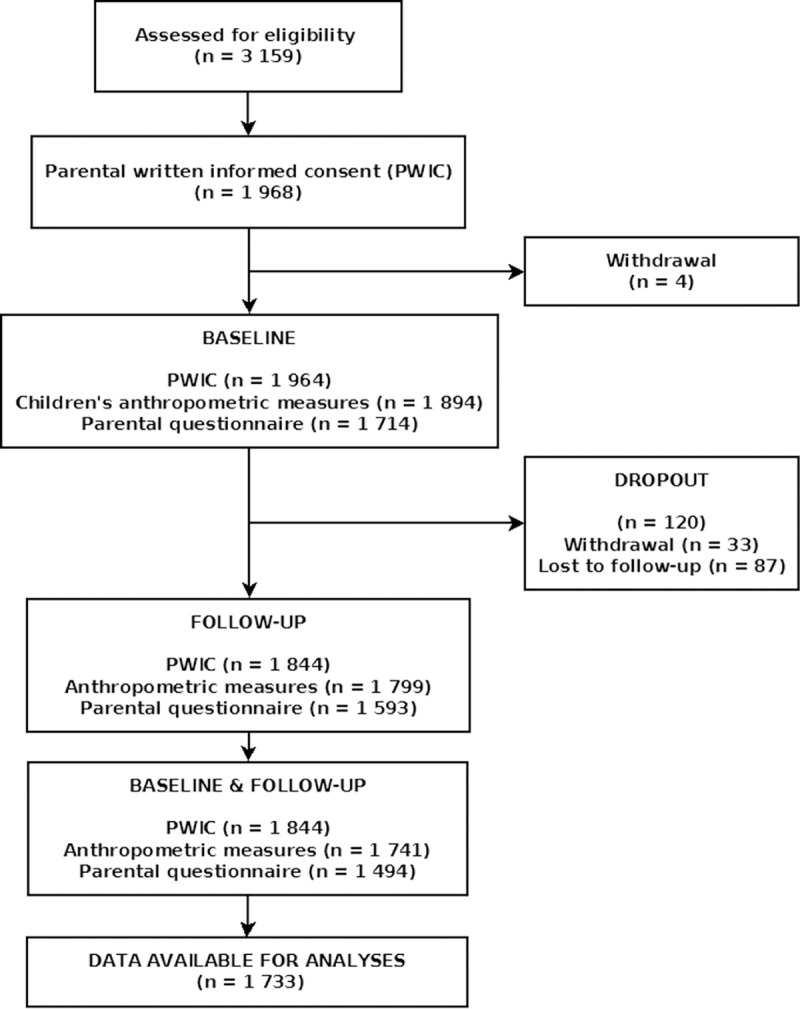
Flowchart of participants/datasets in the Baden-Württemberg Study.

The 32 consulting teachers offered vocational training in their respective regions. Recruitment took place in the academic year 2009/2010, when teachers who applied for these training sessions in the following year were asked to participate in the outcome evaluation. This resulted in 157 classes in 86 schools, which were part of the baseline measurements in 2010, and 154 classes in 84 schools in the follow-up, one year later. In total, 439 teachers participated in those training sessions, of which 81 were engaged in the intervention. The parents of 1968 pupils in grade one and grade two gave their written informed consent. Fig 1 shows a flowchart with the respective numbers of schools, teachers and their classes at each stage of the trial. The corresponding number of participants or available datasets, respectively, is depicted in Fig 2.

### Data collection

Data collection took place at the beginning of the academic year, in fall 2010 (baseline), and fall 2011 (follow-up), respectively. A team of scientific researchers and trained students visited each participating school and carried out the measurements of the children. Parents received self-administered questionnaires.

#### Questionnaires and derived variables

Both parents were asked to specify their educational level and their monthly household income. They were invited to report their height, weight and waist circumference, and whether they were current smokers.

Mothers had to indicate their age at the time of giving birth, whether they had gestational diabetes or smoked during pregnancy, and whether the child was breastfed. A migration status of the child was assumed if at least one parent was born abroad or at least one parent mainly spoke a foreign language during the child’s first years of life. Additionally, single parenthood was assessed. As parameters of the health behaviour of the children, the daily time of outdoor play, the number of days per week they met the WHO guideline of moderate to vigorous physical activity (MVPA) of more than 60 minutes, the amount of screen media consumption, the consumption of sugar sweetened beverages, and breakfast habits were assessed.

Family educational level was ranked in accordance with the CASMIN classification system as the highest level of two parents or the level of a single parent [17]. It was dichotomized for analysis into tertiary level, on the one side, versus primary and secondary level on the other side. Monthly household income was grouped into a low (< €1750) and a high (≥ €1750) category. Outdoor play time was dichotomized into > 60 min/day and ≤ 60 min/day. Reaching the WHO Guideline for MVPA on at least four days a week was compared to reaching it on less than four days. The use of screen media was divided into > 60 min/day and ≤ 60 min/day, and the consumption of sugar sweetened beverages into more than one time per week and one time or less. Having breakfast was divided into “never” and “rarely” vs. “often” and “every day”.

#### Anthropometrics

Anthropometric measurements of the children were taken by trained staff, according to the International Society for the Advancement of Kinanthropometry (ISAK) standards [18]. Weight and height were measured using calibrated flat scales, respectively mobile stadiometers (both by Seca® Company, Germany). Body Mass Index (BMI) was calculated by the quotient of weight in kilograms and height in m^2^ and converted to BMI percentiles using German reference data [19]. WC was measured precisely halfway between the iliac crest and the lower costal border in centimetres using a metal tape measure (Lufkin® Industries Inc., Texas, USA). WHtR was calculated by the ratio of WC and height in centimetres. A WHtR ≥ 0.5 was determined as abdominally obese [20].

Parental weight, height and WC were assessed by the parents in a self-report, and BMI and WHtR were converted by the researchers from the relevant parameters. A parental BMI ≥ 25 was categorized as overweight and a BMI ≥ 30 as obese [21].

#### Assessment of intervention costs

The chosen perspective for the evaluation was a societal one that implies a collection of all costs and consequences incurred during the one year intervention period in 2010/2011 [22]. A bottom up, micro-costing approach was applied with the detailed collection of data of resource use [23]. The costs regarding the development and evaluation of the intervention were not included, only the costs for the delivery of the intervention in a routine manner were assessed at the time of incurrence.

The costs were divided in those of two seminars for each of the 32 consulting teachers, costs of three vocational training sessions for participating teachers, and personnel costs. All costs were assessed and described as accurately and in as much detail as possible.

Costs for seminars included personnel costs for speakers, rent of seminar rooms, subsistence costs, travel expenses and hotel costs for consulting teachers, materials (folders, CDs, copies), letters and envelopes, postal charges, and distributed folders. Costs for vocational training contained copies for both consulting teachers and participating teachers, materials (CDs, files, sheets etc.), subsistence costs, distributed folders, shipping envelopes and postal charges for materials, advertising materials (poster, flyer, brochures), and costs for the process evaluation of the vocational training sessions including post-paid envelopes and postal charges. The personnel costs included the salary of consulting teachers, the secretary and six researchers.

Most of the costs were assessed alongside the intervention, and therefore represent the exact prices and real market prices of the reference year 2011. Some unrecorded costs in the evaluation period, e.g. the exact amount of printed pages for the seminars, were estimated, but close to the original amounts and prices. No labour costs for participating teachers were added to the costs as the intervention was included in the curriculum.

The total costs were calculated by multiplying the quantity and the unit costs for each item. All positions were weighted with the factor 81/439, as 81 out of 439 teachers participated in the evaluation. For the calculation of the costs per child, the entire number of pupils in the intervention classes (n = 1458) was used, assuming that all children benefited from the intervention, independent of their study participation.

No discounting was applied because the period under consideration lasted only one year. No unintended consequences (e.g. side effects or adverse outcomes) or intangible costs were expected. Costs as well as effects concerning the intervention were compared to “doing nothing“, respectively the later start of the intervention in the waiting control group.

#### Calculation of the Incremental Cost-Effectiveness Ratio (ICER

The incremental cost-effectiveness ratio (ICER) is defined as the ratio of net intervention costs and net intervention effects, with C_I_ representing the average costs per participant in the intervention group, and C_C_ the average costs per participant in the control group, which in the present study equals zero:

$$ICER=\frac{C_{I}-C_{C}}{E_{I}-E_{C}}=\frac{\Delta C}{\Delta E}$$

Likewise E_I_ and E_C_ represent the average effects in their respective group, in this study E_I_−E_C_ stands for the number of cases of abdominal obesity averted.

The ICER is calculated for several numbers of participants reached by the intervention. For that purpose, the incidence rates of abdominal obesity of the study population at follow-up (n = 1733) in the intervention (2.2%, 95% CI [1.3; 3.1]) and control group (3.5%, 95% CI [2.2; 4.8]) were applied firstly to the number of participants in the intervention group as intended to treat (n = 1072); secondly as the number available on follow-up (n = 955); thirdly on datasets without missing variables for regression analysis (n = 847); and fourthly for the extrapolation to all children (n = 1458), who belonged to the participating study classes of the intervention group (including those without agreement for study participation). Finally, the above-mentioned incidence rates were used for the extrapolation of cases averted by the estimated number of all children (n = 40000), who were reached by the intervention until the academic year 2013/14.

#### Sensitivity analysis

Since the data for this research was of primary nature and no modelling was performed, sensitivity analyses are restricted. Variables for sensitivity analyses are mainly the respective differences in costs and effects. As no individual different costs per child, teacher or school incurred, there was no variation in costs to enter into a sensitivity analysis. Concerning effectiveness, it seems possible that the reduction in the incidence rate of abdominal obesity in another country may be higher or lower. Therefore, we calculated the ICER for the costs of cases averted at a 10% and 20% higher and lower effect, respectively, on the incidence rate.

#### Losses to follow-up, missing data

Losses to follow-up and missing data are common problems in observational trials, and may bias the results. To examine baseline differences between participants who took part in both measurements and those who were lost to follow-up, the Mann-Whitney-*U* test for continuous data, and Fisher’s exact test for categorical data were used. The same applies for differences between records with complete data for the regression analyses and those who were excluded because of missing explanatory variables. Reasons for losses to follow-up of participating children were family relocation, grade repetition, and sick leave.

#### Statistical analysis

The descriptive analysis of the baseline data included all variables that were considered meaningful for the characterization of the groups and were established, or supposed correlates, of the key values. Differences between intervention and control, as well as differences between weight groups, were tested with respect to the scale level and the underlying distribution with Fisher’s exact test for categorical data and t-test, Mann-Whitney *U* test or Welch-test (considering heterogeneity in variance) for continuous data. Significance level was set to α < 0.05 for two-sided tests. WHtR was multiplied by 10 for the regression analysis to facilitate the interpretability of results, so one unit in the regression model represents 0.10 WHtR.

The adjusted effect for the key value “incidental abdominal obesity” was analysed in a logistic regression with the stepwise selection of all variables from Table 1, except those of children’s anthropometrics. In a further step, the missing values of the predictor “skipping breakfast” were imputed with a random draw from the Bernoulli distribution of the variable, and this was done separately for boys and girls. A potential clustering effect in schools was tested in a two-level model (generalized linear mixed model).

**Table 1:** Baseline characteristics of participants in the Baden-Württemberg Study.

	Missing values	Intervention (n = 955)	Control (n = 778)	Total (n = 1733)
Boys, n (%)		481 (50.4)	372 (47.7)	852 (49.2)
Age, years [m (sd)]		7.09 (0.63)	7.06 (0.63)	7.08 (0.63)
Migration background, n (%)	244	280 (34.2)**	182 (27.2)	462 (31.0)
**Anthropometrics**				
BMIPCT, [m (sd)]		48.92 (27.80)	48.09 (27.48)	48.55 (27.65)
WHtR, [m (sd)]		0.449 (0.038)	0.447 (0.040)	0.448 (0.039)
Overweight incl. obesity, n (%)		96 (10.1)	69 (8.9)	165 (9.5)
Obesity, n (%)		41 (4.3)	27 (3.5)	68 (3.9)
Abdominal obesity, n (%)		80 (8.4)	55 (7.1)	135 (7.8)
**Pregnancy and birth**				
Mother´s age at birth, [m (sd)]	258	30.11 (5.08)	30.76 (5.04)	30.40 (5.07)
Gestational diabetes, n (%)	205	30 (3.6)	31 (4.5)	61 (4.0)
Smoking during pregnancy, n (%)	196	94 (11.2)	62 (8.9)	156 (10.1)
Breastfeeding, n (%)	194	708 (83.6)	578 (83.5)	1286 (83.6)
**Parental characteristics**				
Single parent, n (%)	218	90 (10.8)	66 (9.7)	156 (10.3)
Tertiary family educational level, n (%)	269	268 (33.2)	207 (31.6)	475 (32.4)
Household income < 1750 €, n (%)	381	94 (12.8)	77 (12.4)	171 (12.6)
Overweight/obesity (mother), n (%)	300	247 (31.4)	193 (29.8)	440 (30.7)
Overweight/obesity (father), n (%)	392	463 (62.2)	354 (59.3)	817 (60.9)
Abdominal obesity (mother), n (%)	788	255 (49.6)	192 (44.5)	447 (47.3)
Abdominal obesity (father), n (%)	871	352 (74.9)	290 (74.0)	642 (74.5)
Smoking (mother), n (%)	236	184 (22.4)*	119 (17.6)	303 (20.2)
Smoking (father), n (%)	295	246 (31.2)	172 (26.5)	418 (29.1)
**Health and lifestyle characteristics**				
Playing outside > 60 min/day, n (%)	248	547 (66.5)*	473 (71.3)	1020 (68.7)
MVPA ≥ 4 days/week ≥ 60 min/day, n (%)	263	216 (26.7)	183 (27.7)	399 (27.1)
Screen media > 60 min/daily, n (%)	205	122 (14.5)	83 (12.1)	205 (13.4)
Soft drinks > 1 time/week, n (%)	197	208 (24.6)	156 (22.6)	364 (23.7)
Skipping breakfast, n (%)	195	109 (12.9)	89 (12.9)	198 (12.9)

m (mean), sd (standard deviation)

** p < 0.01

* p < 0.05.

Descriptive and bivariate statistics were conducted with IBM SPSS Release 21.0 for Windows (SPSS Inc, Chicago, IL, USA). Linear and logistic regression models as well as multi-level models to account for the clustering of data were calculated with the statistical software package R Release 3.1.2 for Windows (http://cran.r-project.org).

## Results

### Baseline characteristics

There were some differences between the intervention and control group in the baseline characteristics of participants shown in Table 1. More children with a migration background existed in the intervention group. Moreover, children in the intervention group seemed to be less active in playing outside and their mothers more often smoked. These variables were included in subsequent regression analyses.

### Losses to follow-up, missing data

The analysis of the baseline data for those who participated in the study at both measurements, and those for whom only baseline values were available, showed some differences. Children who participated only in the baseline measurements were more frequently overweight, obese and abdominally obese and had, more often, a migration background. They spent more time with screen media and consumed more soft drinks. Their mothers were younger, smoked more often, and the household income was more frequently below €1750.

The baseline values of participants who were excluded from the regression analyses, due to missing values, differed in some variables from those with complete data. Children with missing values were more frequently overweight, obese and abdominally obese and had a single parent. They spent more time with screen media, consumed more soft drinks and less often had breakfast. Their mothers were younger and had more frequently experienced gestational diabetes. Their fathers were more often smokers. Families had fewer tertiary educational level participants, and household income was more frequently below €1750.There were no differences between the intervention and control group in the numbers of losses to follow-up, and missing data.

### Intervention effects

Bivariate comparisons between the intervention group and controls concerning changes between baseline and follow-up measurements (Table 2) showed significant differences in anthropometric outcome variables, only for BMI percentiles. This difference was examined more closely in a regression analysis. The same applied to the incidence of abdominal obesity where the highest, albeit not statistically significant (*p*-value for a two-sided Fisher’s exact test 0.14) difference, was found.

**Table 2:** Changes baseline—follow-Up.

	Missing	Intervention (n = 955)	Control (n = 778)	Total (n = 1733)	*p*-value
**Anthropometrics**					
BMI percentile, [m (sd)]	5	**0.67 (10.34)**	0.17 (10.17)	0.45 (10.26)	**0.038**
WHtR, [m (sd)]		-0.007 (0.022)	-0.008 (0.022)	-0.008 (0.022)	0.162
Incidence overweight, n (%)	5	29 (3.1)	19 (2.4)	48 (2.8)	0.466
Remission overweight, n (%)	5	12 (1.3)	11 (1.4)	23 (1.3)	0.835
Incidence obesity, n (%)	5	10 (1.1)	5 (0.6)	15 (0.9)	0.440
Remission obesity, n (%)	5	5 (0.5)	4 (0.5)	9 (0.5)	1.000
Incidence abdominal obesity, n (%)		21 (2.2)	27 (3.5)	48 (2.8)	0.140
Remission abdominal obesity, n (%)		10 (1.0)	14 (1.8)	24 (1.4)	0.217

m (mean), sd (standard deviation)

The significant effect for BMI percentiles was lost after controlling for the baseline value in the regression analysis (*p* = 0.403). The final regression model for the incidence of abdominal obesity (Table 3) shows less than half the odds for children in the intervention group to develop abdominal obesity during the period under study. This result was adjusted for grade, gender, baseline value of WHtR, and breakfast habits. No clustering effect in schools was observed. The above mentioned migration background, maternal smoking behaviour, and the playing outside of the children had no significant influence on the outcome.

**Table 3:** Logistic regression models for the incidence of abdominal obesity with and without imputed values for skipping breakfast.

	Primary model		Model with imputation		
	(n = 1538, R^2^ = 0.14^b^)		(n = 1733, R^2^ = 0.11^b^)		
Covariates	OR	95% CI	OR	95% CI	90% CI
Intervention	**0.48**	**[0.25; 0.94]**	0.60	[0.33; 1.09]	**[0.37; 0.99]**
Grade 2	**0.38**	**[0.19; 0.79]**	**0.47**	**[0.25; 0.88]**	**[0.28; 0.80]**
Female	1.19	[0.62; 2.29]	1.40	[0.77; 2.54]	[0.85; 2.31]
WHtR baseline^a^	**4.34**	**[2.39; 7.88]**	**3.39**	**[2.07; 5.56]**	**[2.24; 5.14]**
Skipping breakfast	**3.68**	**[1.85; 7.33]**	**3.03**	**[1.59; 5.79]**	**[1.76; 5.22]**

OR odds ratio, CI confidence interval.

^a^ multiplied by 10.

^b^ Nagelkerke.

To retrieve a model utilizing the complete number of available datasets, the missing values of the variable “skipping breakfast” were imputed. The resulting regression model (Table 3) differed in the proportion of explained variance, and the magnitude of the odds ratios. Particularly, the OR for the intervention group was only significant on the 10% level. Therefore, 90% confidence intervals (CI) are shown additionally in Table 3.

### Costs

An overview of all costs, separately displayed, for seminars for consulting teachers, vocational training sessions for participating teachers, and personnel costs can be found in Table 4. For reasons of clarity, most of the unit costs of materials were combined and presented as one price. A more detailed table providing the costs of each item assessed separately can be found in S1 Table.

**Table 4:** Costs for the intervention in the year 2010/2011 in Euro.

Category	Quantity	Unit costs	Total costs	Weighted *
**Two seminars for consulting teachers**				
Personnel costs for speakers	1	260.00	260.00	47.97
Rent of seminar rooms	1	372.80	372.80	68.79
Subsistence costs	1	2812.87	2812.87	519.00
Travel expenses	1	3560.12	3560.12	656.88
Hotel costs	1	2713.50	2713.50	500.67
Materials (folders, CDs, copies)	1	634.36	634.36	117.05
Letters and envelopes	1	44.48	44.48	8.21
Postal charges	1	88.00	88.00	16.24
Distributed folders for consulting teachers	32	38.90	1244.80	229.68
**Three vocational training sessions (only for intervention teachers)**				
Copies for consulting teachers	2592	0.12	298.08	55.00
Copies for teachers	10975	0.12	1262.13	232.88
CDs, files, sheets etc.	1	264.35	264.35	48.78
Subsistence costs	32	200.00	6400.00	1180.87
Number of distributed folders	439	38.90	17077.10	3150.90
Shipping envelopes for consulting teachers for vocational training materials	1	15.84	15.84	2.92
Postal charges for shipping envelopes and packets	1	257.70	257.70	47.55
Advertising materials (poster, flyer, brochures)	1	5913.41	5913.41	1091.09
**Process evaluation of vocational trainings**				
Post-paid envelopes and postal charges for consulting teachers	1	78.08	78.08	14.41
Post-paid envelopes and postal charges for teachers	1	258.12	258.12	47.63
**Personnel costs **				
Salary of consulting teachers	29	1200.00	34800.00	6420.96
	1	700.00	700.00	129.16
Secretary (50% of working time used for intervention)	1 x 75%-position (1*37.5%)	40800.00	15300.00	2823.01
Researcher (50% of working time used for intervention)	1 x 75%-position 1 x 100%- position 4 x 50%-position (1*37.5%, 1*50%, 4*25%)	55200.00	103500.00	19096.81
**Total**			197855.74	36506.41
**Per child (n = 1458)**				25.04

* for 81 intervention teachers out of a total of 439 teachers in vocational trainings (81/439).

*Note*. A more detailed table providing the costs of each item assessed separately can be found in S1 Table.

The costs for two seminars for the consulting teachers added up to €2164.48, with the highest amount of money spent on the teachers´ accommodation, travel and catering. The three vocational training sessions added up to €5872.00, the distributed folders for all teachers, advertising materials and the catering were the most expensive positions. The personnel costs contained costs for consulting teachers and the staff of the university and were, in total, the highest position at €28469.93. The total amount of the intervention for one year was €36506.41, which resulted in an intervention cost of €25.04 per pupil.

### Costs per case averted

The ICERs, in this scenario the costs per case of incidental abdominal obesity averted, varied between €1515 and €1993, depending on the size of the observed target group. Table 5 shows calculations for different numbers of participants reached by the intervention program.

**Table 5:** Different model calculations for costs per case of abdominal obesity averted.

	Cases expected	Cases observed	Cases averted	Total costs	Costs / case averted
IG follow-up complete DS in logistic regression (n = 847)	30 ^b^	16	14	847 * €25.04	€1514.92
IG follow-up (n = 955)	33 ^b^	21	12	955 * €25.04	€1992.77
IG baseline (n = 1072)	38 ^b^	24 ^a^	14	1072 * €25.04	€1917.35
All pupils in the intervention classes (n = 1458)	51 ^b^	32 ^a^	19	€36506.41^c^	€1921.39
All pupils, who were approximately reached until the academic year 2013/14 (n = 40000)	1400 ^b^	880 ^a^	520	40000 * €25.04	€1926.15

IG intervention group, CG control group, DS data sets, CI confidence interval.

^a^ Incidence rate IG (n = 955): 0.022; 95% CI [0.013; 0.031].

^b^ Incidence rate CG (n = 778): 0.035; 95% CI [0.022; 0.048].

^c^ see Table 4.

### Sensitivity analysis

Hypothetical changes in the effect of the intervention on the incidence rate of ±10% and ±20% respectively, resulted in a minimum of costs per case averted of €1789.53 and a maximum of €1963.92.

## Discussion

### Interpretation of findings in relation to other literature

Despite the large amount of interventions and studies focusing on the prevention of obesity in children, it is difficult to compare them with the present study. Studies often lack detailed information about the intervention components and how the intervention is implemented [10, 24, 25]. Concerning cost-effectiveness, this limited transparency aggravates the transferability to other settings and interventions [26]. Additionally, cost-effectiveness studies in general, and especially those of good quality, are scarce, [10, 24, 26]. Finally, the huge variety of outcome measures and the predominance of modelling studies exacerbate the difficulty of comparability.

Frequently used outcome measures are BMI and BMI-related measures. To the authors’ knowledge, no intervention studies evaluating the cost-effectiveness of the prevention of incidental abdominal obesity in children are available in literature. Most studies using modelling report costs per Quality Adjusted Life Years (QALY) or Disability Adjusted Life Years (DALY). However, within public health settings QALYs are difficult to apply, and the focus should rely on a much broader range of outcomes [27]. Especially complex public health interventions require measures that go beyond QALYs, e.g. non-health related outcomes such as education [27]. Furthermore, in primary prevention the target group is expected to be in good health, so QALYs are difficult to assess as they focus on disease. Lastly, there is some doubt concerning the validity of QALYs in pediatric economic evaluation [28]. Reviews focusing on cost-effectiveness analysis for health promotion programs for children demonstrated that few studies were just primary data studies and that a large amount of the studies were modelling studies [11, 25, 26, 29]. Because the latter are difficult to compare to the present study, the focus in this section is on primary data studies. John et al. 2010 identified twelve cost-effectiveness studies aimed at the prevention of paediatric obesity; of which ten were modelling studies and two primary data studies [25]. In the first primary data study, Wang et al. calculated for their “FitKid” after school program, per capita net intervention costs of $317 per student who attended; ≥ 40% of the intervention [30]. In the second primary data study, McAuley et al. assessed for their community-based APPLE-Project about NZ$641 per child per year [31]. For the update of their review in 2012, John et al. detected further economic evaluations of interventions, 10 with a model approach and one primary data study [11]. For the primary data study, Moya Martinez et al. [32] reported costs of about €270 per child per year for their after school care program and, calculated by John et al. [11], saved €500 per % point decrease in triceps skinfold thickness. Korber identified in her review eight studies which used a modelling approach and four which conducted cost-effectiveness analysis in the school setting, including the above mentioned Wang et al. and McAuley et al. [26]. The third study, Kesztyüs et al., determined the costs of the URMEL-ICE intervention, the previous incarnation of the present project, as €11.11 per cm WC- and €18.55 per unit WHtR-gain prevented [12]. The fourth study of Krauth et al. calculated €619 per student per year for their PA intervention [33]. Lobstein [29] identified 21 cost-effectiveness studies, with only three based on primary data, ranked as likely to be cost effective and already described above [30, 31, 32].

In sum, the costs of the present intervention at €25.04 per child per year are less than the reported costs of many other studies. Purchasing power parity estimates for the year 2011 enable comparisons of health costs between different countries[34]. Accordingly, after correction to inflation, the “FitKid” program would cost €218.94, the “Apple” project €391.05 and finally the “Cuenca study” of Moya Martinez and collegues €277.67 per child and year (all costs in 2011 Euros). However, Wang et al. and McAuley et al. confirm personnel as the most expensive category [30, 31]. After all, drawing conclusions about transferability, comparing interventions which used external personnel instead of training teachers, interventions conducted in different countries with different school systems and which used different outcome measures, is nearly impossible. Additionally, none of those studies provided a detailed list with all costs incurred in order to create a qualitative comparison.

### Context of findings

The first school-based program initiated by Ulm University (URMEL-ICE) was successful in terms of cost-effectiveness, but it was limited to the manageable area of Ulm [12]. A broader approach was started with the “Join the Healthy Boat” health promotion program, which was designed to cover the entire state of Baden-Württemberg, southern Germany [13]. The exciting question was whether the program would be successful on such a large scale, and whether it would prove to be cost-effective. In this article, we have demonstrated the effectiveness of state-wide health promotion of abdominal obesity and reported the costs in total, per capita, and per case of abdominal obesity averted.

Abdominal obesity was a primary target for several reasons. Firstly, because obesity-related health risks are explained by waist circumference (WC), not BMI [35]; secondly because WC is sensitive towards changes in physical activity [36] and nutrition [37, 38], both components of the intervention; thirdly because BMI fails to identify obesity in a significant percentage of children [39]; and finally in the light of increasing rates of abdominal obesity in children [4, 5].

Childhood obesity very likely continues into adulthood [6, 7]. Our data shows a high prevalence of abdominal obesity in parents (47% in mothers, 75% in fathers), although we have to rely on self-reported measures by the parents. Moreover, when social desirability and underreporting are considered, the real prevalence might even be higher. Realizing the close relationship between abdominal obesity and metabolic syndrome [40], the strongest risk factor for cardio-metabolic diseases that represent the main causes of death in the developed world, the urgency for counteractive measures is obvious. Accordingly, an early start to health promotion and prevention is adequate and important. The settings of school and kindergarten offer a great opportunity to reach the major part of an important and vulnerable age group. Additionally, the involvement of social and family components, especially the inclusion of parents in an intervention, is important and has an impact on becoming overweight and obese [24]. Finally, health status and education, e.g. the capacity to learn, are related and have an important influence on later life [10].

Fighting abdominal obesity will not only have an impact on the burden of disease posed by dietary and activity related NCDs, but will also help to cut the costs of obesity. Direct costs of obesity are estimated at €22.4 billion in Germany in 2020, plus indirect costs of €3.3 billion through losses in productivity [41]. In the United States, obesity-attributable medical costs for non-institutionalized adults were estimated at $190.2 billion, or 20.6 percent of national health expenditure in 2005 [42]. Additionally, childhood obesity is responsible for $14.1 billion in direct medical costs [43]. In this context, costs of €1515 up to €1993 per case of averted abdominal obesity are a good and prudent investment. Moreover, costs per child and year of €25.04 are completely covered by the parental willingness to pay (WTP) €123 per year, assessed within the Baden-Württemberg Study and described in detail elsewhere [44].

Costs can be kept down with a teacher-driven intervention in regular lessons. This bottom-up principle is less cost consuming than a top-down expert driven intervention, because e.g. no additional costs for working time and travel incur. The costs for the intervention material itself were very limited (€38.90) and the folder is reusable any number of times for further school classes. Furthermore, once a teacher is trained, no further costs arise and teachers themselves may instruct colleagues at their school in using the materials, thus saving the costs of vocational training sessions.

The analyses of losses to follow up and missing values showed that those individuals were of specific interest as they showed higher risk behaviours for becoming overweight and obese. Therefore, it is necessary for future research to focus on the reduction of participants´ dropout rates to ensure the inclusion of vulnerable groups. However, no effect of migration was seen when included in the regression analysis.

### Strengths and limitations

A strength of the present study is its cluster-randomized intervention trial design; its application throughout the complete state of Baden-Württemberg, and the collection of the data of nearly 2000 children and their parents. Furthermore, the particular costs of the intervention are directly assessed and not estimated. To the authors’ knowledge, no other study has provided such a comprehensive and detailed list of all costs incurred as the present study has. Additionally, trained staff measuring the children´s anthropometrics ensures high quality and using WHtR instead of other anthropometrics such as BMI is indispensable when investigating abdominal obesity in children, the latter being a more suitable definition of obesity in children. The advanced statistical analyses are further evidence of high methodological quality.

A strength of the school-based intervention, “Join the Healthy Boat”, is its aim to address the main health behaviours influencing abdominal obesity, namely physical activity, diet, and media consumption. The bottom-up approach, with teachers as enactors of the intervention, as well as the “train-the-trainer” and “peer-to-peer” concept, has several advantages compared to a top-down and expert-driven approach. Firstly, teachers training teachers implies a relationship of equals; the consulting teachers are aware of obvious obstacles and opportunities, and the trained teachers discuss more openly. Secondly, teachers can embed the intervention in their school-materials, implying constant utilization. Thirdly, teachers maintain contact with parents on a regular basis resulting in the higher involvement of parents in the intervention. Fourthly, as already mentioned, costs can be saved as no costs for personnel arise. A high valuation and appreciation of the intervention by teachers, as well as parents, can be inferred from the regular contact and discussion between teachers and the research team, as well as from the high response rate of parents returning the questionnaires.

A limitation of the present study is the voluntary participation of teachers that may involve only motivated teachers who already have a positive attitude towards health and healthy behaviour, and probably already include those topics in their classes. Furthermore, practically no “high risk schools” were present in this study. Another source of selection bias may occur at the parental level. Parents, especially those with little knowledge of the German language, may answer questionnaires only partly, or they may completely refrain from participation. Furthermore, baseline differences between the intervention and control group arose. To account for those imbalances, adequate adjustment was part of all statistical analyses. Another common problem of observational studies are the missing values and losses to follow-up that may lessen the precision of the results, therefore missing data analyses were performed and imputation considered. Lastly, questionnaires can be the source of socially desired answers and have the Hawthorne effect; the latter defined as participants behaving differently knowing they are being observed.

External validity and generalizability require the study population to be a subset of the target population. In our case, the rates of overweight and obesity observed in the participants of the present study do not differ from data that have been identified for Baden-Württemberg in a survey in German schoolchildren [45].

### Implications

The health promotion program “Join the Healthy Boat” has now been extended to kindergarten, aimed at children between three and six years, including their parents. Considering the high proportion of parents with abdominal obesity, from 47% in mothers up to 75% in fathers, this program focuses exactly on the right topic. An outcome evaluation of the program in kindergarten, including an economic evaluation, will follow.

## Conclusions

Prevention and health promotion, as important parts of health care, should be available to all citizens on equal terms. Prevention of abdominal obesity is of particular importance because of its association with most of the non-communicable diseases (NCD). Therefore, an early start to health promoting measures needs to be implemented. Settings like school and kindergarten are especially suitable because most of the children can be easily reached through simple and inexpensive means. Costs of €25.04 per child and year are justifiable regarding the parental willingness to pay [44], and could even be reduced by integrating key elements of health promotion in the training of teachers, and including the present program in the school curriculum. Although the training of teachers and input into the school curriculum are not part of the medical sector, it is advisable in the context of “Health in all Policies” [46] to invest cross-sectoral to improve public health. In view of the apparent need for action, in regard to the increasing health risks posed by abdominal obesity and its sequelae [47], the authors recommend the transfer of scientific results as presented in this study into political decision making.

## Supporting information

S1 Checklist CONSORT 2010 Checklist. (DOC)

S1 Protocol Study Protocol of the Baden-Württemberg Study. (PDF)

S1 Table Detailed description of costs. (PDF)
