# Supplementary material for: Costs and effects of a state-wide health promotion program in primary schools in Germany – the Baden-Württemberg Study: A cluster-randomized, controlled trial
Source: PLoS One. 2017 Feb 21;12(2):e0172332. doi: 10.1371/journal.pone.0172332 (PMC5319648; doi:10.1371/journal.pone.0172332)
Supplement: S1 Protocol — (PDF) [file pone.0172332.s002.pdf]

ulm university universität uulm

Studienplan für die Evaluationsstudie zur Untersuchung der Wirksamkeit der Intervention im Projekt „Komm mit in das gesunde Boot – Grundschule“

DRKS-ID: DKRS00000494

Inhalt

1. Darstellung des aktuellen Standes der Forschung 2
2. Zielstellung der Studie und erwarteter klinischer Nutzen 3
3. Art und Design der Studie 3
4. Definition der Beobachtungseinheit und der Ein- und Ausschlusskriterien 4
5. Beschreibung der Intervention 5
6. Aufnahmeverfahren und Gruppenzuteilung 5
7. Definition der Zielgrößen 6
8. Darstellung des Untersuchungsprogramms 7
9. Bewertung der Wirksamkeit 8
10. Bewertung der Sicherheit 8
11. Fallzahlplanung und statistische Auswertung 8
12. Ethische Aspekte, Regulatorien 8
13. Vereinbarungen zur Publikation der Ergebnisse 9
14. Teilnehmer und Verantwortlichkeiten, Finanzierung der Studie 9
15. Unterschriften 10
16. Literatur 11

Anhang 13

1. Darstellung des aktuellen Standes der Forschung

Die Problematik hoher Raten von Übergewicht bei Kindern ist seit langem bekannt. Die aktuellsten Zahlen, die für eine repräsentative Stichprobe von Kindern und Jugendlichen in Deutschland vorliegen, gehen von 15,4% bei der Altersgruppe der 7-10jährigen aus, bei den 11-14jährigen sind es bereits 18,6% (Kurth & Schaffrath, 2007). Neben einer genetischen Prädisposition (Hebebrand & Hinney, 2009) sind es vor allem Faktoren des Lebensstils, die für die ansteigenden Prävalenzen von Übergewicht und Adipositas verantwortlich gemacht werden: geringe körperliche Aktivität (Andersen et al., 2006) und eine übermäßige Kalorienzufuhr (Olstad & McCargar, 2009). Verschiedene Umweltbedingungen fördern diese ungünstige Entwicklung bei Kindern.

Doch auch das Ess- und Ernährungsverhalten normalgewichtiger Kinder entspricht in vielen Fällen nicht den Ansprüchen an eine ausgewogene Ernährung: Ein leicht zugängliches Überangebot hochkalorischer Nahrungsmittel (Süßigkeiten, Limonaden, Fast Food) verbunden mit einer Etablierung ungesunder Essgewohnheiten (ohne Frühstück in die Schule gehen, Süßigkeiten als Pausenbrot) prägen das Bild der Ernährung von Schulkindern: 30,7% der Jungen und 21,8% der Mädchen von 7-10 Jahren trinken ein- oder mehrmals täglich Softdrinks. Nur 53,6% der Jungen und 60,6% der Mädchen derselben Altersgruppe essen mindestens einmal täglich frisches Obst (Mensink, Kleiser & Richter, 2007).

Parallel zu den Veränderungen im Ernährungsbereich hat sich in den letzten Jahrzehnten der Alltag von Kindern zunehmend bewegungsarmer gestaltet. Rund ein Viertel der Kinder treibt keinen regelmäßigen Sport (Lampert et al., 2007). In vielen Fällen hat zu Fuß gehen oder Radfahren als Möglichkeit, von einem Ort zum anderen zu kommen, ausgedient. Die Gelegenheiten für Kinder, ihre Freizeit im Sitzen zu verbringen, nehmen durch die Verfügbarkeit und die Beliebtheit von Fernsehern, Computern und Videospielen zu: 80% der 6-13jährigen Kinder sehen jeden oder fast jeden Tag fern (Feierabend & Rathgeb, 2007). Nicht in allen Gegenden sind zudem ausreichend Spielflächen und -möglichkeiten sowie Sport- oder andere sinnvolle Freizeitaktivitäten für Kinder vorhanden.

Angesichts der hohen Beeinträchtigungen von Kindern mit Übergewicht oder Adipositas im gesundheitlichen (Baker et al., 2007) und psychosozialen Bereich (Schwimmer et al., 2003) und der oft mangelhaften Effizienz von Therapiemaßnahmen (Latzer et al., 2009) sind Präventionsmaßnahmen dringend erforderlich.

Ein Umfeld, das besonders für langfristige und flächendeckende Präventionsmaßnahmen geeignet scheint, ist das Setting Schule (Pyle et al., 2006). Mit nur geringem Aufwand können sehr viele Kinder erreicht werden. Änderungen, die sich in der Schulumwelt und in den Unterrichtsinhalten und -methoden bewähren, können auch für nachfolgende Schülerjahrgänge genutzt werden. Kinder mit besonderem Risiko für oder mit bereits bestehendem Übergewicht lassen sich oft nur schwer für Maßnahmen zur Gesundheitsförderung gewinnen. Deswegen bietet sich ein Interventionsansatz an, der vom Lehrer ausgehend die gesamte Klasse mit einbezieht.

Die Anzahl nachgewiesen wirksamer Präventionsprogramme ist aktuell vergleichsweise niedrig (Summerbell et al., 2005; Stice et al., 2006). Gründe können beispielsweise ungeeignete Methoden, zu kurze Interventionszeiträume, methodische Probleme, wie das Fehlen eines randomisierten kontrollierten Studiendesigns, ungenügende statistische Power oder ungeeignete Zielgrößen sein (Kropski, Keckley & Jensen, 2008).

Wesentliche Elemente der Studie „Komm mit in das gesunde Boot – Grundschule“ basieren auf der Studie URMEL-ICE (“Ulm Research on Metabolism, Exercise, and Lifestyle Intervention in Children”, Nething et al., 2006, Nagel et al., 2009), einer randomisierten kontrollierten Studie zur Untersuchung der Wirksamkeit eines Programms zur Prävention von Übergewicht im Kindesalter. In der Studie erfolgte eine einjährige schulbasierte Intervention mit den Schwerpunkten Förderung der Bewegung, Reduktion des Konsums zuckerhaltiger Getränke und des Medienkonsums. Das Studienkollektiv bestand aus 1427 Schülern aus 64 Klassen in 32 Schulen der Region Ulm, Alb-Donau-Kreis und Neu-Ulm. Ziel der Studie war die Prävention von Übergewicht bei Grundschulkindern durch eine schulbasierte Intervention, die auf langfristige Änderung des Gesundheitsverhaltens unter Einbeziehung von Familie und Freunden abzielt. Die

URMEL-ICE Intervention, die ohne zusätzliche Unterrichts- und Lehrerstunden auskommt, wurde in enger Zusammenarbeit mit einer Gruppe von Grundschullehrern entwickelt und von der Mehrzahl der Lehrer wie auch von Schülern und Eltern sehr positiv aufgenommen. Im Ergebnis der Studie zeigten sich positive Effekte in der Interventionsgruppe, u.a. eine verminderte Inzidenz bei parallel gesteigerter Remission von Übergewicht, eine Abnahme der Körperfettmasse und eine Verbesserung der motorischen Leistungsfähigkeit.

Einordnung der Evaluationsstudie:

Das Projekt „Komm mit in das gesunde Boot – Grundschule" (finanziert von der Baden-Württemberg Stiftung) ist ein umfassendes Programm zur Gesundheitsförderung, welches durch die Universität Ulm durchgeführt und wissenschaftlich begleitet wird. Kern des Projekts ist ein schulbasiertes Programm, bei dem Grundschulkinder über den Lehrer bei der Entwicklung eines gesunden Lebensstils in Bezug auf Ernährung, Bewegung und Freizeitgestaltung unterstützt werden sollen. Dies geschieht schuljahresbegleitend mithilfe speziell entwickelter Materialien für den Regelunterricht (Wartha & Brandstetter, 2009) sowie unter Einbeziehung der Eltern. Die Inhalte des Programmes lehnen sich an die Vorgaben des Bildungsplanes für den Fächerverbund MeNuK an. Ziel ist die Steigerung der körperlichen Aktivität der Grundschulkinder, die Reduktion des Konsums zuckerhaltiger Getränke sowie die Verringerung des Medienkonsums. Die landesweite Verbreitung des Programms erfolgt über ein Multiplikatorensystem: Für jeden der 44 Landkreise in Baden-Württemberg werden Grundschullehrer als Multiplikatoren ausgebildet, die wiederum die Fortbildung von weiteren Lehrern in ihrer Region realisieren.

Im Rahmen des Programms soll in einer randomisierten zweiarmigen longitudinalen Studie die Wirksamkeit der Intervention untersucht werden (Evaluationsstudie).

2. Zielstellung der Studie und erwarteter klinischer Nutzen

Zur Untersuchung der Wirksamkeit der Intervention wird die Veränderung von Merkmalen gemessen, die verschiedene Bereiche betreffen (siehe Abschnitt 5).
Im Mittelpunkt steht die Frage, ob sich präventionsrelevante Gesundheitsparameter durch ein schulbasiertes Programm positiv beeinflussen lassen.
Erwartet wird eine positive Veränderung des Gesundheitsverhaltens in den Bereichen Ernährung, Bewegung und Freizeitverhalten bei den Schülern der Interventionsgruppe im Vergleich zur Kontrollgruppe sowie eine Verbesserung der auf diese Verhaltensweisen bezogenen Gesundheitsparameter.

3. Art und Design der Studie

Die Evaluationsstudie ist eine prospektive zweiarmige randomisierte longitudinale Studie, in welcher die schulbasierte einjährige Intervention (Interventionsgruppe, IG) mit einer Gruppe verglichen wird, in der keine Intervention erfolgt (Kontrollgruppe, KG).
Die Messungen erfolgen zu zwei Zeitpunkten: Jeweils zu Beginn der Schuljahre 2010/2011 (Baseline-Erhebung) und 2011/2012 (Follow-Up-Erhebung), wobei mit ‚Beginn' die Zeitspanne vom Beginn des Schuljahres bis zum Beginn der Herbstferien gemeint ist. Es wird das sogenannte Wartekontrollgruppendesign angewendet, d.h. in der einen Gruppe von Klassen wird das Programm im Schuljahr 2010/2011 durchgeführt (Interventionsgruppe), die andere Gruppe von Klassen (Kontrollgruppe) erhält im Schuljahr 2010/2011 keine Intervention und startet mit dem Programm erst im Schuljahr 2011/2012, d.h. nach einem Jahr Wartezeit.
Zu beiden Zeitpunkten werden Merkmale erhoben, die folgende Bereiche betreffen:

- Körperliche, emotionale und geistige „Fitness" des Kindes
- Gesundheitsbezogene Lebensqualität des Kindes
- Verhalten und verhaltensbezogene Kognitionen des Kindes und seiner Eltern

- Aktivitätsverhalten des Kindes
- Soziodemographie
- Anamnese von Eltern und Kind
- Veränderungen im Unterricht und in der Schulumgebung
- Gesundheitsökonomie

Die Evaluationsstudie bezieht sich auf Klassen des Anfangsunterrichtes (1. und 2. Jahrgangsstufe). Die Randomisierung in die beiden Gruppen erfolgt schulbasiert (siehe Abschnitt 6).

Für die Beobachtungseinheit in der Evaluationsstudie, dem einzelnen Kind, ergibt sich wegen der Cluster-Struktur (bedingt durch die schulbasierte Randomisierung) eine komplexere Datenstruktur (die Verschachtelung „Kind innerhalb einer Schule"), was bei der statistischen Auswertung berücksichtigt werden muss (siehe Abschnitte 9 und 11). Eine verblindete Durchführung der Studie ist nicht möglich.

Die Interventionsmaßnahmen werden über die Dauer eines Schuljahres ausgeführt. Die folgende Abbildung zeigt das Ablaufschema nach der Randomisierung, beginnend mit dem Schuljahr 2010/2011:

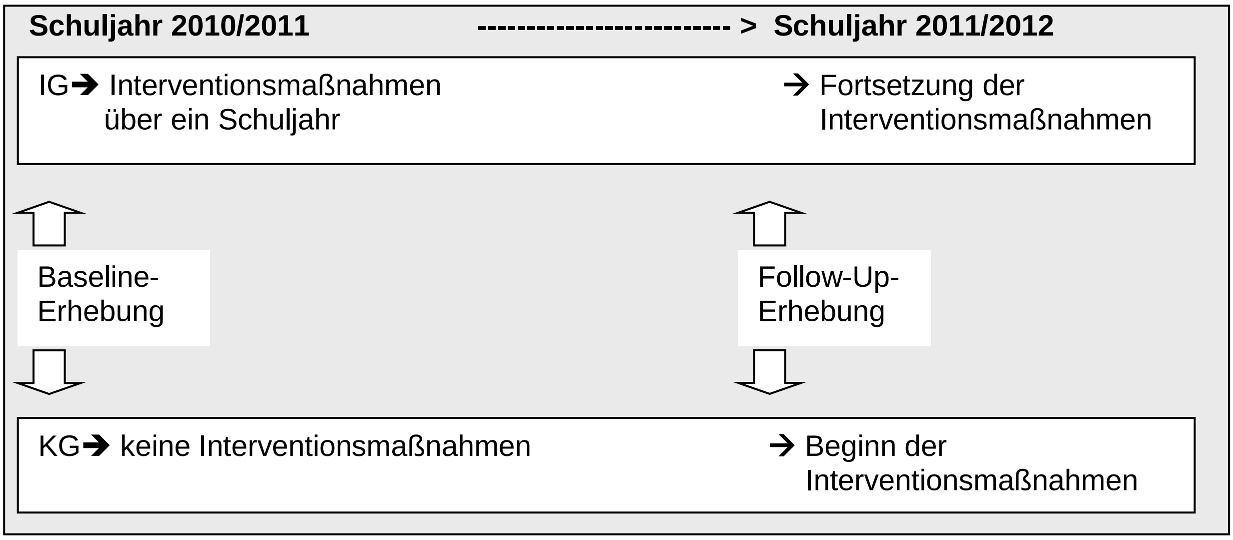

Als Zeitraum der Untersuchungen ist jeweils der Beginn der Schuljahre 2010/2011 und 2011/2012 vorgesehen:
Baseline-Erhebung: 7 Wochen (13.09.2010 – 29.10.2010)
Interventionsphase: Schuljahr 2010/2011
Follow-Up-Erhebung: 7 Wochen (12.09.2011 – 28.10.2011)

Organisation der Studie:
Die Geschäftsstelle und die Studienzentrale befinden sich in den Räumen des Arbeitsbereiches der Projektgruppe „Komm mit in das gesunde Boot - Grundschule", Haus 58/33 Frauensteige 6, 89075 Ulm. Das Datenzentrum ist im Institut für Biometrie, Schwabstraße 13, 89075 Ulm.
Für die Messungen zu den beiden Erhebungszeitpunkten werden regionale Messteams gebildet und im Rahmen einer Schulung auf die spezifischen Aufgaben vorbereitet.

4. Definition der Beobachtungseinheit und der Ein- und Ausschlusskriterien

Die zu randomisierenden Klassen haben folgende Einschlusskriterien zu erfüllen.

- Die Lehrkraft muss im Schuljahr 2009/2010 die entsprechende Fortbildung des Programms "Komm mit in das gesunde Boot – Grundschule" erhalten haben oder im Schuljahr 2010/2011 daran teilnehmen (für die Interventionsgruppe).
- Die Lehrkraft muss sich für die Teilnahme am Programm (inklusive Randomisierung) im Schuljahr 2010/2011 angemeldet haben (für die Kontrollgruppe).
- Eine Einverständniserklärung der teilnehmenden Lehrkraft muss vorliegen.

Ausschlusskriterien sind:

- Von Seiten der Schule wird keine Möglichkeit gegeben, die zur Evaluation nötigen Erhebungen durchzuführen.

Randomisiert werden nur Klassen, in denen das Programm im Schuljahr 2010/2011 erstmals durchgeführt wird. Da die Beobachtungseinheit das einzelne Kind ist, ist für die Evaluationsstudie die Einverständniserklärung der Eltern erforderlich. Unabhängig von der Einverständniserklärung der Eltern nehmen aber alle Kinder der Interventionsgruppe am Programm teil.

5. Beschreibung der Intervention

Die Intervention ist eine kombinierte verhaltens- und verhältnispräventive Maßnahme und hat die drei folgenden verhaltensbezogenen Ziele:
1.) mehr körperliche Aktivität,
2.) weniger Konsum von zuckerhaltigen Getränken,
3.) weniger Konsum von Bildschirmmedien.
Ein wichtiger Gesichtspunkt ist die Einbeziehung der Familie. Grundlegender Unterschied zu anderen Programmen ist die Integration in den regulären Schulablauf: Die Intervention ist Teil des Unterrichts bzw. der Pausen, zusätzliche Schulsportstunden sind nicht vorgesehen.
Die Intervention wird vom Klassenlehrer anhand eines speziell ausgearbeiteten Manuals (Wartha & Brandstetter, 2009) nach Teilnahme an einer dreiteiligen regionalen Fortbildung durchgeführt. Vorgesehen sind zwei schultägliche Bewegungseinheiten von je 5 bis 7 Minuten sowie 20 Unterrichtseinheiten zu den Themen „Getränke“, „Bewegung“, „Freizeitgestaltung“ im Verlauf des Schuljahres. Um die Eltern in das Programm mit einzubeziehen, sind Vorlagen für Elternabende und -briefe sowie sog. Familien-Hausaufgaben vorgesehen.
In der Kontrollgruppe finden keine Interventionsmaßnahmen statt, die Lehrkräfte haben noch keine Fortbildung erhalten, haben aber Interesse an der Fortbildung.

6. Aufnahmeverfahren und Gruppenzuteilung

Die Randomisation erfolgt schulbasiert. Als Randomisationseinheiten dienen Grundschulen in Baden-Württemberg, die Untersuchungseinheiten sind die Grundschüler.
Für die Interventionsgruppe kommen diejenigen Klassen in Frage, in denen im Schuljahr 2010/2011 die Intervention erstmals durchgeführt wird. Die Lehrkräfte dieser Klassen haben bereits im Schuljahr 2009/2010 die entsprechende Fortbildung des Programms „Komm mit in das gesunde Boot - Grundschule“ erhalten oder werden im Schuljahr 2010/2011 daran teilnehmen. In die Kontrollgruppe können diejenigen Klassen randomisiert werden, die bis jetzt nicht am Programm teilgenommen haben und deren Lehrkräfte noch keine Fortbildung erhalten haben, aber Interesse an der Fortbildung im Schuljahr 2010/2011 haben. Die Zuteilung in die Kontrollgruppe erfolgt als Zufallsauswahl aller Lehrkräfte, die sich für die Teilnahme im Schuljahr 2010/2011 am Programm anmelden.
Die folgende Abbildung zeigt schematisch die Zuteilung der Lehrer zu den Gruppen IG und KG der Evaluationsstudie:

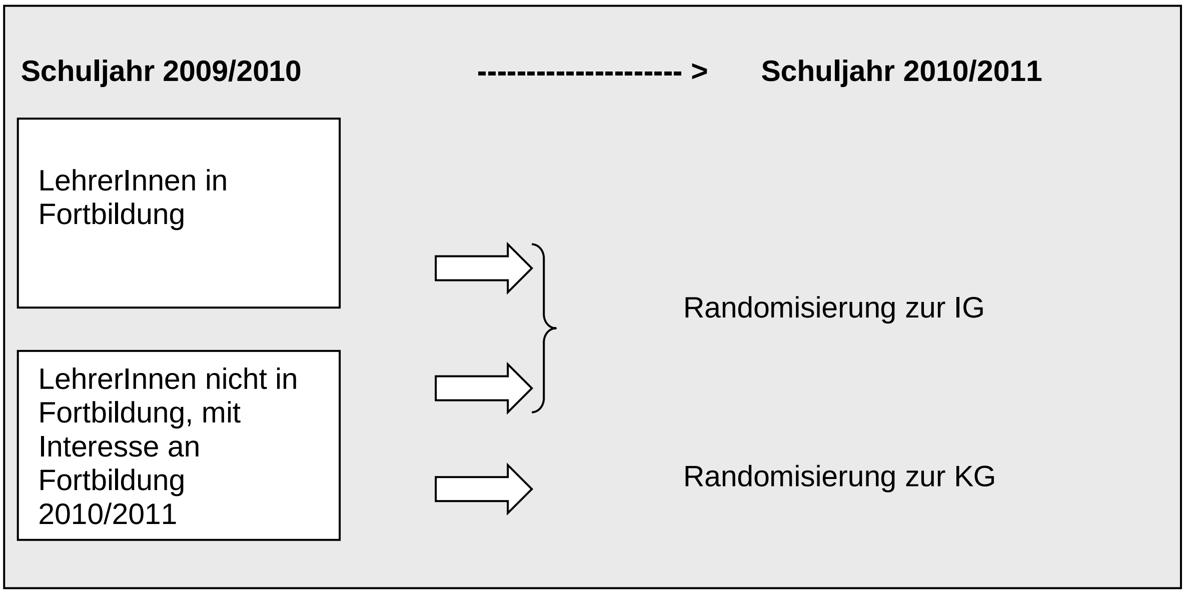

Die Randomisation erfolgt als Blockrandomisation. Ziel ist es, Gruppen annähernd gleicher Größe hinsichtlich Zahl der Schüler und Zahl der Klassen zu schaffen. Die Randomisationsliste wird vom Institut für Biometrie von einem nicht an dem Projekt beteiligten Mitarbeiter erstellt. Die Randomisation wird mit einer Spezialsoftware durchgeführt (Randomisationssoftware ROM, Rohlmann et al., 2004).

7. Definition der Zielgrößen

Primäre Zielgrößen sind Reduktionen in den altersbedingten Zuwächsen in den anthropometrischen Parametern

- subscapulare Hautfaltendicke,
- Bauchumfang und
- Ausdauerleistungsfähigkeit (zurückgelegte Strecke im 6-Minuten-Lauf) als Differenz von Follow-Up-Messung und Baseline-Messung.

Sekundäre Zielgrößen sind die Veränderungen (Differenz von Follow-Up-Messung und Baseline-Messung) folgender Bereiche:

- anthropometrische Parameter (BMI, Hautfaltendicke Triceps)
- gesundheitsbezogene Lebensqualität des Kindes (KINDL-R, Eltern-Version, Ravens-Sieberer, 2003)
- sportmotorische Fähigkeiten des Kindes (Testbatterie DKT zur Erfassung der Leistungsfähigkeit der motorischen Hauptbeanspruchungsformen, Dordel & Koch, 2004)
- kognitive Leistungen des Kindes (DL-KE, Kleber & Kleber, 1974; Testbatterie KiTAP zur Aufmerksamkeitsprüfung von Kindern, Zimmermann et al. ,2003)
- emotionales Verhalten des Kindes (Children`s Behavior Questionnaire, CBQ, Putnam & Rothbart, 2006; Befragung)
- Ernährung des Kindes (Items im Elternfragebogen)
- Physische Aktivität/ Energieverbrauch des Kindes (Items im Elternfragebogen und einachsige Bewegungssensoren Actiheart mit synchroner Erfassung der Herzfrequenz, Brage et al., 2004)
- Medienkonsum des Kindes (Items im Elternfragebogen)
- Schulumwelt (Fragebogen für die Lehrkraft)

In Abhängigkeit der Skala der Zielgrößen kommen für die statistische Auswertung der primären und sekundären Zielgrößen geeignete statistische Verfahren in Frage, die eine adäquate Berücksichtigung der Clusterstruktur der Daten ermöglichen, vergleiche Abschnitte 3, 9 und 11.

Da derzeit die Testphase für einzelne Erhebungsinstrumente noch nicht abgeschlossen ist, können an dieser Stelle noch keine Details genannt werden.

8. Darstellung des Untersuchungsprogramms

Die folgende Tabelle gibt einen Überblick über die zu beiden Zeitpunkten zu erhebenden Parameter und die Art ihrer Erhebung:

**Parameter**	**Baseline-Messung**	**Follow-Up-Messung**
Zeitpunkt	Herbst 2010	Herbst 2011
Ein-, Ausschlusskriterien	x	
Einverständniserklärung (Eltern und Lehrer)	x	
Elternfragebogen:	x	x
Anamnese des Kindes	x	x
Ernährung des Kindes	x	x
Physische Aktivität des Kindes	x	x
Medienkonsum des Kindes	x	x
Anamnese der Eltern	x	x
Medienkonsum der Eltern	x	x
Physische Aktivität der Eltern	x	x
Selbstwirksamkeit und Beziehung der Eltern zum Kind	x	x
Soziodemographische Merkmale der Eltern	x	
Einschätzung der Lebensqualität des Kindes (KINDL-R)	x	x
Einschätzung des emotionalen Verhaltens des Kindes (CBQ)	x	x
Kinderfragebogen:	x	x
Emotionales Wohlbefinden, Verhalten, verhaltensbezogene Kognitionen (selbstentwickelte Items)	x	x
Messung der kognitiven Fähigkeiten (DL-KE, KiTAP)*	x	x
Körperliche Untersuchung des Kindes, anthropometrische Parameter (Körperhöhe, -gewicht, Bauchumfang, Hautfaltendicke Triceps, subscapular)	x	x
Sportmotorische Untersuchung (DKT)	x	x
Aktivitätsmessung mit Bewegungssensoren (Actiheart)*	x	x
Lehrerfragebogen: Schulumwelt	x	x

Der Elternfragebogen wird über den Klassenlehrer an die Eltern weitergegeben und vom Klassenlehrer in verschlossenen Kuverts auch wieder eingesammelt und ungeöffnet an die Studienzentrale geleitet. Die Schulumwelt wird vom Klassenlehrer beurteilt. Das Ausfüllen des Kinderfragebogens, Messungen der kognitiven Fähigkeiten sowie körperliche Untersuchungen und sportmotorische Tests werden im Rahmen von regulären Schulstunden von den regionalen Messteams vor Ort ausgeführt (siehe Abschnitt 3). Zur Untersuchung der Aktivität der Kinder ist der Einsatz von Bewegungssensoren vorgesehen.
Die mit * markierten Parameter werden nur in einer Teilstichprobe untersucht.
Da derzeit die Testphase für einzelne Erhebungsinstrumente noch nicht abgeschlossen ist, können an dieser Stelle noch keine Details zu einzelnen Messinstrumenten genannt werden. Dies wird zu einem späteren Zeitpunkt ergänzt.

9. Bewertung der Wirksamkeit

Primäre Zielgrößen sind subscapulare Hautfaltendicke, Bauchumfang und zurückgelegte Strecke im 6-Minuten-Lauf als Differenz von Follow-Up-Messung und Baseline-Messung. Zur Untersuchung der Wirksamkeit sollen gemischte lineare Regressionsmodelle angewendet werden, mit den Einflussgrößen Gruppenzugehörigkeit und weiteren prognostischen Variablen (siehe Abschnitt 11). Die Wirksamkeit kann dann anhand von Größe und Lage des Konfidenzintervalls für die Gruppendifferenz beurteilt werden.

10. Bewertung der Sicherheit

Alle Interventionsinhalte vertiefen Themen, die im Bildungsplan des Landes Baden-Württemberg für den Anfangsunterricht (Jahrgansstufe 1 und 2) vorgesehen sind. Die Inhalte werden während des regulären Schulunterrichtes vom zuständigen Lehrer vermittelt und unterliegen damit seiner Verantwortung.
Alle Untersuchungen sind weitgehend risikofrei. Durch geschultes und erfahrenes Personal im Umgang mit Kindern sollen diese Risiken minimiert werden.

11. Fallzahlplanung und statistische Auswertung

Im Anhang sind Ergebnisse von Simulationen einiger möglicher Szenarien für die Powerabschätzung der Studie enthalten. Aus Gründen der Machbarkeit wird von zwei Klassengrößen (20 und 30 Schüler) und drei möglichen Anzahlen von Klassen ausgegangen (25, 40 und 50 Klassen pro Gruppe). Die minimale Anzahl beträgt 1000 Schüler (2x25 Klassen mit je 20 Schülern), im maximalen Fall wird von 3000 Schülern (2x50 Klassen mit je 30 Schülern) ausgegangen. Das Gesamtsignifikanzniveau wird auf 0.05 festgelegt. Bei der Powerabschätzung wurde wegen der drei gleichberechtigten primären Zielgrößen (siehe Abschnitt 7) eine Adjustierung für multiples Testen nach Bonferroni vorgenommen (alpha=0.05/3), die Ergebnisse basieren auf zweiseitigen Tests. In Abhängigkeit von der Anzahl der Schulen und der Klassengröße kann eine Power von

- 52% bis 93% (Differenz in subscapularer Hautfaltendicke),
- 61% bis 97% (Differenz in Bauchumfang) und
- 49% bis 90% (Differenz in gelaufener Strecke im 6-Minuten-Lauf)

erreicht werden.
Wegen der Cluster-Struktur der Daten erfolgt die Auswertung der primären und sekundären Zielgrößen mit adäquaten statistischen Methoden. Zu diesen Verfahren gehören lineare gemischte Modelle für die primären Zielgrößen (Verbeke & Molenberghs, 2009), GEE Modelle für ordinale oder nominale Zielgrößen (Zeger & Liang, 1986). Bei der Auswertung ist zu berücksichtigen, dass neben der Gruppenzugehörigkeit weitere potentielle Einflussgrößen wie beispielsweise Migrationshintergrund, Stadt/Land oder Bildungsstand der Eltern in die Analysen einbezogen werden müssen. Die Auswertung der Studie erfolgt in Anlehnung an den Intention-to Treat (ITT)-Ansatz, es erfolgt aber keine Ersetzung von fehlenden Werten. Zur Untersuchung der Stabilität der konfirmatorischen Datenanalyse ist eine Sensitivitätsanalyse geplant (Ersetzung fehlender Werte mittels multipler Imputationsverfahren).
Wegen der drei gleichberechtigten primären Zielgrößen erfolgt eine Adjustierung nach Bonferroni (alpha=0.05/3).
Zur Untersuchung von Effekten in speziellen Teilkollektiven (z.B. Kinder mit Migrationshintergrund, übergewichtige Kinder) sind zusätzlich explorative Untergruppenanalysen geplant.

12. Ethische Aspekte, Regulatorien

Die Evaluationsstudie wird in Übereinstimmung mit der Deklaration von Helsinki und unter Beachtung der ICH-GCP Guidelines durchgeführt. Die Teilnahme der Kinder und ihrer Eltern an

der Studie ist freiwillig und kann jederzeit ohne Angabe von Gründen und ohne Nachteile für Kinder, Eltern oder Lehrer zurückgezogen werden. Es werden die Bestimmungen des Bundesdatenschutzgesetzes eingehalten.

13. Vereinbarungen zur Publikation der Ergebnisse

Für die Publikation der Studienergebnisse gelten die in der Geschäftsordnung vom Februar 2010 genannten Vereinbarungen, sowie die im Vertrag zwischen der Baden-Württemberg Stiftung und dem Universitätsklinikum Ulm genannten Abmachungen vom November 2009 (siehe Anlage Kopie der Geschäftsordnung).

14. Teilnehmer und Verantwortlichkeiten, Finanzierung der Studie

Studienleitung:
Prof. Dr. Jürgen Michael Steinacker, Sektion Sport- und Rehabilitationsmedizin, Universitätsklinikum Ulm, Steinhövelstraße 9, 89070 Ulm
Weitere StudienmitarbeiterInnen der Sektion Sport- und Rehabilitationsmedizin: Susanne Brandstetter (Dipl.-Psychologin), Susanne Kobel (Dipl.-Sportwissenschaftlerin), Dr. Benjamin Koch (Dipl.-Sportwissenschaftler), Dmytro Prokopchuk (Dipl.-Sportwissenschaftler), Anja Schreiber (Dipl.-Psychologin), Olivia Wartha (Medienpädagogin M.A.), Sarah Weber (Dipl.-Sportwissenschaftlerin), Tamara Wirt (Dipl.-Psychologin)

Weitere beteiligte Einrichtungen:
Institut für Psychologie und Pädagogik (Leitung: Prof. Dr. Tina Seufert)
Albert-Einstein-Allee 47, 89081 Ulm
Weiterer Studienmitarbeiter des Instituts für Psychologie: Dr. Uwe Fischer (Dipl.-Psychologe)

Institut für Biometrie (komm. Leitung: apl. Prof. Dr. Rainer Muche)
Schwabstr. 13, 89075 Ulm
Weitere StudienmitarbeiterInnen des Instituts für Biometrie: Dr. Jens Dreyhaupt (Dipl.-Mathematiker), Sabrina Sufeida (Medizinische Dokumentation u. Informatik, B.Sc.), Martina Wiedom (Dipl.-Statistikerin)

Das Projekt „Komm mit in das gesunde Boot-Grundschule“ und auch die hier beschriebene Evaluationsstudie werden von der Baden-Württemberg Stiftung finanziert.

15. Unterschriften

Ich habe den Studienplan gelesen und bin einverstanden mit der Durchführung der Studie im Einklang mit diesem Studienplan:

Sektion Sport- und Rehabilitationsmedizin:

Prof. Dr. Jürgen M. Steinacker (Projektleiter)	Ulm
Name (in Druckbuchstaben)	Ort
Datum	Unterschrift

Institut für Psychologie und Pädagogik:

Prof. Dr. Tina Seufert	Ulm
Name (in Druckbuchstaben)	Ort
Datum	Unterschrift

Institut für Biometrie:

Prof. Dr. Rainer Muche	Ulm
Name (in Druckbuchstaben)	Ort
Datum	Unterschrift

16. Literatur

Andersen LB, Harro M, Sardinha LB et al. (2006). Physical activity and clustered cardiovascular risk in children: a cross-sectional study (The European Youth Heart Study). *Lancet, 368(9532):299-304.*

Baker JL, Olsen LW, & Sorensen TI (2007). Childhood body-mass index and the risk of coronary heart disease in adulthood. *New England Journal of Medicine, 357,* 2329-2337.

Brage S, Brage N, Franks P, Ekelund U, Wong M, Anderson L, Froberg K & Wareham N (2004). Branched equation modelling of simultaneous accelerometry and heart rate monitoring improves estimate of directly measured physical activity energy expenditure. *Journal of Applied Physiology,96(1):343-51.*

Dordel S, Koch B (2004): Basistest zur Erfassung der motorischen Leistungsfähigkeit von Kindern und Jugendlichen. Deutsche Sporthochschule Köln. [http://www.chilt.de/Inhalt/Manual_DKT.pdf], Zugriff am 17.05.10.

Feierabend S, Rathgeb T (2007). KIM-Studie 2006 – Kinder und Medien. Computer und Internet. Basisuntersuchung zum Medienumgang 6- bis 13-Jähriger in Deutschland. Stuttgart: Medienpädagogischer Forschungsverbund Südwest (Hrsg.).

Hebebrand J, Hinney A (2009). Environmental and genetic risk factors in obesity. *Child Adolesc Psychiatr Clin N Am, 18(1):83-94.*

Kleber EW. & Kleber G (1974). *Differentieller Leistungstest - KE (DL-KE). Handanweisung*. Göttingen: Verlag für Psychologie Dr. C. J. Hogrefe.

Kurth B-M & Schaffrath Rosario A (2007). Die Verbreitung von Übergewicht und Adipositas bei Kindern und Jugendlichen in Deutschland. *Bundesgesundheitsblatt Gesundheitsforschung Gesundheitsschutz, 50, 736-743.*

Kropski JA, Keckley PH, Jensen GL (2008). School-based obesity prevention programs: an evidence-based review. *Obesity Research, 16(5):1009-18.*

Lampert T, Mensink GB, Romahn N, Woll A (2007). Physical activity among children and adolescents in Germany. Results of the German Health Interview and Examination Survey for Children and Adolescents (KiGGS). *Bundesgesundheitsblatt Gesundheitsforschung Gesundheitsschutz, 50(5-6):634-42.*

Latzer Y, Edmunds L, Fenig S, Golan M, Gur E, Hochberg Z et al. (2009). Managing childhood overweight: behavior, family, pharmacology, and bariatric surgery interventions. *Obesity, 17, 411-423.*

Mensink GB, Kleiser C, Richter A (2007). Food consumption of children and adolescents in Germany. Results of the German Health Interview and Examination Survey for Children and Adolescents (KiGGS). *Bundesgesundheitsblatt Gesundheitsforschung Gesundheitsschutz, 50(5-6):609-23.*

Nagel G, Wabitsch M, Galm C et al. (2009). Determinants of obesity in the Ulm Research on Metabolism, Exercise and Lifestyle in Children. *European Journal of Pediatrics,* 68(10):1259-67

Nething K, Stroth S, Wabitsch M et al. (2006) [Primary prevention of sequelae of adiposity in children and adolescents]. *Deutsche Zeitschrift für Sportmedizin, 57(2):42-5.*

Olstad DL, McCarcar L (2009). Prevention of overweight and obesity in children under the age of 6 years. *Applied Physiology Nutrition and Metabolism, Aug;34(4):551-70.*

Putnam S P & Rothbart MK (2006). Development of short and very short forms of the Children's Behavior Questionnaire. *Journal of Personality Assessment, 87(1)*:103-113.

Pyle SA, Sharkey J, Yetter G, Felix E, Furlong MJ, Poston WSC (2006). Fighting an epidemic: the role of schools in reducing childhood obesity. *Psychology in Schools, 43(3):361-76.*

Ravens-Sieberer U (2003). Der Kindl-R-Fragebogen zur Erfassung der gesundheitsbezogenen Lebensqualität bei Kindern und Jugendlichen - Revidierte Form. In J.K.A.Schumacher & E. Brähler (Eds.), *Diagnostische Verfahren zu Lebensqualität und Wohlbefinden* (pp. 184-188). Göttingen: Hogrefe.

Rohlmann F, Muche R, Goldschmidt L, Goldschmid R. Das Randomisationsprogramm ROM Handbuch Version 2.02, Stand: Juli 2004

Schwimmer JB, Burwinkle TM, & Varni JW (2003). Health-related quality of life of severely obese children and adolescents. *JAMA, 289,* 1813-1819.

Summerbell CD, Waters E, Edmunds LD, Kelly S, Brown T, Campbell KJ (2005). Interventions for preventing obesity in children. *Cochrane Data-base Syst Rev, CD001871.*

Stice E, Heather S, & Marti CN (2006). A meta-analytical review of obesity prevention programs for children and adolescents: The skinny on interventions that work. *Psychological Bulletin, 132,* 667-691.

Verbeke G & Molenberghs G (2009). *Linear Mixed Models for Longitudinal Data*. Springer Verlag.

Wartha O & Brandstetter S (2009). *Komm mit in das gesunde Boot – Grundschule. Bewegung, Ernährung und Freizeitgestaltung mit den Inselpiraten – 1./2. Klasse*. Donauwörth: Auer Verlag GmbH.

Zeger SL & Liang KY (1986). Longitudinal Data Analysis for Discrete and Continuous Outcomes. *Biometrics, 42: 121–130.*

Zimmermann P, Gondan M & Fimm B. (2003). *Testbatterie zur Aufmerksamkeitsprüfung für Kinder (KiTAP).* Herzogenrath: Psytest.

Anhang

Powerabschätzung für die Evaluationsstudie
im Projekt „Komm mit in das gesunde Boot-Grundschule“

Grundlage:
Daten von der URMEL-Ulm Studie. Für diese Daten wurde ein gemischtes Regressionsmodell mit ‚Schule’ als zufälligen Effekt und der Gruppenzugehörigkeit als festen Effekt angepasst, um die Varianz und die Regressionskoeffizienten zu schätzen. Mit den geschätzten Regressionskoeffizienten wurden neue Studiendaten für verschiedene Szenarien simuliert. Das entsprechende gemischte Regressionsmodell wurde für die ermittelten Varianzen an den neu erzeugten Datensatz angepasst und für jedes Szenario die Power aus dem entsprechenden Test für den Gruppenunterschied (Kontrast für feste Effekte im gemischten Regressionsmodell) berechnet. Es wurde nur die Gruppenzugehörigkeit als Einflussgröße betrachtet, weitere potentielle prognostische Faktoren fanden keine Berücksichtigung.
Weitere Parameter für die Powerabschätzungen: zweiseitiger Test, alpha=0.05.

Anzahl Schüler pro Schule:
Die Anzahl der Schüler pro Schule entspricht dem Median (etwa 30) und Mittelwert (etwa 35) bei URMEL Ulm. Eine Unterteilung in verschiedene Klassen pro Schule wurde nicht vorgenommen.
Die kursiven Zeilen in der umseitigen Tabelle entsprechen der im Studienplan dargestellten Situation mit 2x75 Schulen und jeweils 20 Schülern pro Klasse.

Zielgröße	Anzahl Schulen	Anzahl Schüler pro Schule	Gesamtstichprobenumfang	Power(%)
diff_subsc	2x25	30	1500	77
	2x25	35	1750	79
	2x40	30	2400	93
	2x40	35	2800	94
	2x50	30	3000	97
	2x50	35	3500	98
	2x75	30	4500	100
	2x75	35	5250	100
	2x75	***20***	***3000***	***99***
diff_waist_cu	2x25	30	1500	85
	2x25	35	1750	87
	2x40	30	2400	97
	2x40	35	2800	98
	2x50	30	3000	99
	2x50	35	3500	99
	2x75	30	4500	100
	2x75	35	5250	100
	2x75	***20***	***3000***	***100***

diff_subsc = Differenz in der Hautfaltendicke subscapular
diff_waist_cu= Differenz im Bauchumfang
