# Supplementary material for: Costs and effects of a state-wide health promotion program in primary schools in Germany – the Baden-Württemberg Study: A cluster-randomized, controlled trial
Source: PLoS One. 2017 Feb 21;12(2):e0172332. doi: 10.1371/journal.pone.0172332 (PMC5319648; doi:10.1371/journal.pone.0172332)
Supplement: S1 Table — (PDF) [file pone.0172332.s003.pdf]

Cost-effectiveness of the statewide health promotion program "Join the Healthy Boat" in primary schools

S1: Detailed overview of all costs.

Category	Quantity
Number of participating consulting teachers in the school year 2010/2011	32
Number of participating teachers in the school year 2010/2011	439
Numbers of participating teachers in evaluation schools 2010/2011	81

Consulting teachers (n=32) trained 439 teachers in total in the school year 2010/2011, of which 81 were participating in the evaluation.

The total costs were calculated by multiplying the quantity and the unit costs for each item. As the costs for the delivery of the intervention in a routinely manner were documented within the present study, the total costs were multiplied with the factor 81/439, because 81 teachers out of 439 teachers participated in the evaluation. For the calculation of the costs per child, the entire number of pupils in the intervention classes (n=1458) was used, assuming that all children benefited from the intervention, independent of their study participation.

Note. Numbers are rounded, meaning that differences in sums are due to those rounding differences.

Category	Quantity	Unit costs	Total costs	Weighted (*81/439)	Comments
**Seminars**					
**Seminar 1**					
**Organisation**					
Rent of seminar rooms	1	372.80	372.80	68.79	
Subsistence costs	1	571.80	571.80	105.50	Subsistence on the first day
Subsistence costs	1	612.87	612.87	113.08	Subsistence on the second day
Travel expenses consulting teachers	1	2267.27	2267.27	418.33	All travel expenses are added up to one amount
Hotel costs consulting teachers	1	1633.50	1633.50	301.40	All hotel costs are added up to one amount
**Materials**					
Materials printed double sided and in colour	2400	0.12	276.00	50.92	Per consulting teacher: 75 double sided pages
File	32	0.24	7.68	1.42	Per consulting teacher: 1 file
CDs	64	0.24	15.35	2.83	Per consulting teacher: 2 CDs
CD-labels	64	0.21	13.30	2.45	Per consulting teacher: 2 CD-labels
CD-cases	64	0.05	2.94	0.54	Per consulting teacher: 2 CD-cases
Transparent envelope	32	0.06	1.92	0.35	Per consulting teacher: 1 transparent envelope
**Seminar 2**					
Personnel costs for guest speaker	1	260.00	260.00	47.97	
**Organisation**					
Subsistence costs	1	1628.20	1628.20	300.42	Subsistence for two days
Travel expenses consulting teachers	1	1292.85	1292.85	238.54	All travel expenses are added up to one amount
Hotel costs consulting teachers	1	1080.00	1080.00	199.27	All hotel costs are added up to one amount

Category	Quantity	Unit costs	Total costs	Weighted (*81/439)	Comments
**Materials**					
Printed pages, files, CDs, CD-labels, CD-cases, transparent envelopes	1	317.18	317.18	58.52	Exact the same items and costs as for seminar 1
**Both seminars**					
**Materials**					
Letters of invitation for seminars and salary bills	128	0.05	6.40	1.18	Per consulting teacher: 2 invitations and 2 bills
Christmas card for consulting teachers	32	1.00	32.00	5.90	Per consulting teacher: 1 card
Envelopes	160	0.04	6.08	1.12	Per consulting teacher: 5 envelopes
Postal charges	160	0.55	88.00	16.24	Per consulting teacher: 5x postal charges
Distributed folders including all intervention materials for consulting teachers	32	38.90	1244.80	229.68	Per consulting teacher: 1 folder
**Vocational training sessions**					
Travel expenses consulting teachers	/				Travel expenses are payed by the "Landesinstitut für Schulsport, Schulkunst und Schulmusik Baden-Württemberg"
Travel expenses participating teachers	/				Travel expenses are payed by the "Landesinstitut für Schulsport, Schulkunst und Schulmusik Baden-Württemberg"
**Vocational training 1**					
**Materials**					
Materials printed double sided and in colour for consulting teachers	1024	0.12	117.76	21.73	Per consulting teacher: 32 double sided pages
Materials printed double sided and in colour for teachers	3512	0.12	403.88	74.52	Per teacher: 8 double sided pages

Category	Quantity	Unit costs	Total costs	Weighted (*81/439)	Comments
CDs	64	0.24	15.35	2.83	Per consulting teacher: 2 CDs
CD-labels	64	0.21	13.30	2.45	Per consulting teacher: 2 CD-labels
CD-cases	64	0.05	2.94	0.54	Per consulting teacher: 2 CD-cases
Separator sheet	32	0.03	0.90	0.17	Per consulting teacher: 1 separator sheet
"Pirate game" printed A1	32	7.19	230.08	42.45	Per consulting teacher: 1 printed "pirate game"
**Vocational training 2**					
**Materials**					
Materials printed double sided and in colour for consulting teachers	672	0.12	77.28	14.26	Per consulting teacher: 21 double sided pages
Materials printed double sided and in colour for teachers	1756	0.12	201.94	37.26	Per teacher: 4 double sided pages
Separator sheet	32	0.03	0.90	0.17	Per consulting teacher: 1 separator sheet
**Vocational training 3**					
**Materials**					
Materials printed double sided and in colour for consulting teachers	896	0.12	103.04	19.01	Per consulting teacher: 28 double sided pages
Materials printed double sided and in colour for teachers	5707	0.12	656.31	121.10	Per teacher: 13 double sided pages
Separator sheet	32	0.03	0.90	0.17	Per consulting teacher: 1 separator sheet
**All three vocational training sessions**					
**Organisation**					
Subsistence flat charge for consulting teachers for subsistence of teachers at all three vocational training sessions	32	200.00	6400.00	1180.87	

Category	Quantity	Unit costs	Total costs	Weighted (*81/439)	Comments
**Materials**					
Shipping envelopes for consulting teachers for vocational training session materials (70% of the materials)	66	0.24	15.84	2.92	70% of the vocational training session materials are send as "Maxibrief" (≙ 22 envelopes); 3 envelopes per consulting teacher, one per vocational training session
Postal charges for shipping envelopes for consulting teachers (70% of the materials)	66	2.20	145.20	26.79	70% of the vocational training session materials are send as "Maxibrief" (≙ 22 envelopes); 3x postal charges per consulting teacher
Postal charges packet for consulting teachers (30% of the materials)	30	3.75	112.50	20.76	30% of the vocational training session materials are send as packet (≙ 10 packets); 3x postal charges per consulting teacher, one per vocational training session
Shipping envelope for consulting teachers for sending back the process evaluation questionnaires	32	0.24	7.68	1.42	Per consulting teacher: 1 shipping envelope
Postal charges of post-paid shipping envelopes for consulting teachers	32	2.20	70.40	12.99	Per consulting teacher: 1x postal charges
Envelopes for teachers for sending back the process evaluation questionnaires	439	0.04	16.67	3.08	Per teacher: 1 envelope
Postal charges of post-paid envelopes	439	0.55	241.45	44.55	Per teacher: 1x postal charges
Distributed folders for teachers	439	38.90	17077.10	3150.90	Per teacher: 1 folder
**Public relations**					
"Boots-Tour" travel expenses participating teachers	1	973.20	973.20	179.57	46 teachers participated; costs are added up to one amount

Category	Quantity	Unit costs	Total costs	Weighted (*81/439)	Comments
"Boots-Tour" subsistence costs	1	204.97	204.97	37.82	Subsistence costs for consulting teachers; only 8 submitted their costs to receive the monetary reward
"Boots-Tour" travel expenses research assistants	2494	0.25	623.50	115.04	Distance in km from Ulm to the ten locations of the Boots-Tour of 2011; amount per km: 0.25€
Cover letter	0.5	543.83	271.92	50.17	The assessed costs hold for two years, indicating that only half of the costs are counted
Envelopes C5	0.5	240.40	120.20	22.18	See above
Inlay sheet	0.5	460.77	230.38	42.51	See above
Flyer	0.5	1423.28	711.64	131.30	See above
Poster A3	0.5	462.97	231.48	42.71	See above
Layout poster and flyers	0.5	1492.26	746.13	137.67	See above
Advertisement	1	1800.00	1800.00	332.12	
**Personnel costs**					
Salary of consulting teachers	29	1200.00	34800.00	6420.96	Only 30 salary strokes for 32 consulting teachers: two consulting teachers are sharing the work with someone else, therefore they share their salary
Salary of consulting teachers	1	700.00	700.00	129.16	See above
1 secretary, 50% of total working time (one person; 75% of employment)	50% of total working time	40800.00	15300.00	2823.01	Salary according to “Personalsätze DFG 2010”

Category	Quantity	Unit costs	Total costs	Weighted (*81/439)	Comments
6 research assistants, 50% of total working time (1x75%, 1x100%, 4x50% of employment)	50% of total working time: 1 x 75% 1 x 100% 4 x 50% (1*37.5%, 1*50%, 4*25%)	55200.00	103500.00	19096.81	Salary according to “Personalsätze DFG 2010”
**Total, €**			197855.75	**36506.41**	
**Per pupil (1458)**			135.70	**25.04**	
